# Relevant Choices Affecting the Fatigue Analysis of Ni-Ti Endovascular Devices

**DOI:** 10.3390/ma16083178

**Published:** 2023-04-18

**Authors:** Francesca Berti, Alma Brambilla, Giancarlo Pennati, Lorenza Petrini

**Affiliations:** 1 Department of Chemistry, Materials and Chemical Engineering “G. Natta” (LaBS), Politecnico di Milano, 20133 Milan, Italy; francesca.berti@polimi.it (F.B.); giancarlo.pennati@polimi.it (G.P.); 2 Department of Civil and Environmental Engineering, Politecnico di Milano, 20133 Milan, Italy; alma.brambilla@polimi.it

**Keywords:** in vitro, test environment, test frequency, surrogate specimen, in silico, reliable model, constitutive model, fatigue criterion

## Abstract

Ni-Ti alloys are widely used for biomedical applications due to their superelastic properties, which are especially convenient for endovascular devices that require minimally invasive insertion and durable effects, such as peripheral/carotid stents and valve frames. After crimping and deployment, stents undergo millions of cyclic loads imposed by heart/neck/leg movements, causing fatigue failure and device fracture that can lead to possibly severe consequences for the patient. Standard regulations require experimental testing for the preclinical assessment of such devices, which can be coupled with numerical modeling to reduce the time and costs of such campaigns and to obtain more information regarding the local state of stress and strain in the device. In this frame, this review aimed to enlighten the relevant choices that can affect the outcome of the fatigue analysis of Ni-Ti devices, both from experimental and numerical perspectives.

## 1. Introduction

Nowadays, nickel–titanium (Ni-Ti) alloys, belonging to the family of shape-memory alloys (SMA), are widely used in many application fields, including the aeronautical, structural, and biomedical fields. The fortune of these materials lies in their ability to recover their original configuration even after large stress-induced deformations [1]. This possibility can be microstructurally explained through a phase transformation between two solid lattice structures: austenite, which is stable at temperatures greater than a threshold (T > Af, the austenite finish temperature), and martensite, which is stable at temperatures lower than Mf (the martensite finish temperature), which both depend on the alloy composition and ad hoc thermal treatments. The macroscopic effects of this transformation appears as two different phenomena: pseudoelasticity (PE, also known as superelasticity), which can be exploited at T > Af and consists of the ability to recover stress-induced high deformations (up to 7–8%) by simply removing the load; and the shape-memory effect (SME), which consists of the ability to recover high residual deformations (up to 7–8%) mechanically induced at T < Mf only by increasing the temperature above Af. These particular behaviors come together with other interesting characteristics, such as [2] kinking resistance, good mechanical properties, biocompatibility, thermo-mechanical hysteresis, and good fatigue resistance. Accordingly, Ni-Ti alloys have been widely exploited by the biomedical industry for the development of smart devices that can assume a closed configuration during deployment in the body and an open configuration after implantation, thus enabling minimally invasive surgery. In particular, PE is exploited in peripheral and carotid stents for the treatment of atherosclerosis (a pathological narrowing of the vessel) as well as in prosthetic heart valve frames for the treatment of valvular stenosis or insufficiency. This work is focalized on these devices.

**Figure 1:**
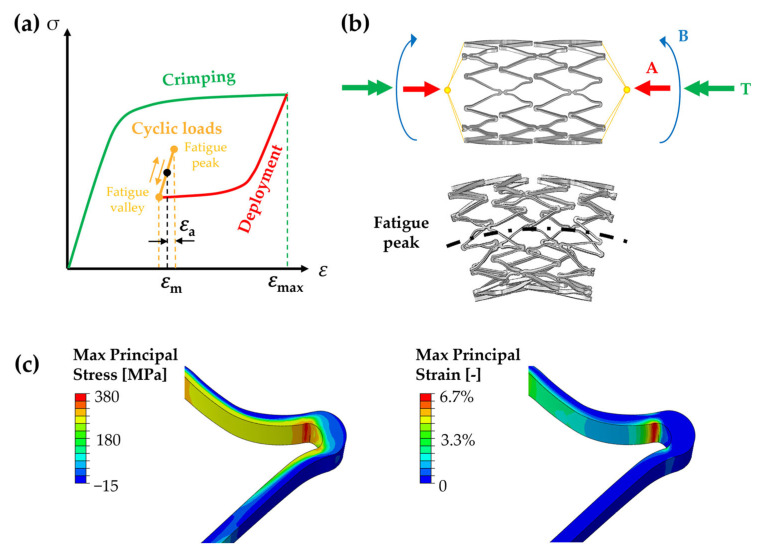
(**a**) Loading sequence of a stent during in vivo implantation and working cycles: the stent reaches a maximum strain, ε_max_, during the crimping phase, it partially self-expands during the deployment into the stenotic artery, and it undergoes cyclic loads defined by a mean strain, ε_m_, and a strain amplitude, ε_a_. (**b**) Schematic representation of the deformation fashion of a peripheral stent under loads representative of an in vivo scenario, including axial compression (A), bending (B), and torsion (T), adapted with permission from [14] (Elsevier, 2023). (**c**) Maximum principal stress and strain distributions in the most-loaded area of the stent at the fatigue peak, showing a gradient in the cross-section.

Stents and valve frames are tubular meshes with diameters ranging between 5–8 mm to 20–35 mm, respectively. Different designs are available on the market, but, typically, these devices present rings made of v-shaped struts and are connected by links with cross-sections in the orders of 0.2 mm × 0.2 mm for stents and up to 0.4 mm × 0.4 mm for valve frames. Due to these similar characteristics, in the following valve frames will also be indicated with the term “stents”. A stent is generally laser-cut from a Ni-Ti tube with a diameter smaller than the final dimension of the device; it undergoes multiple thermal treatments and forming procedures to reach the final expanded configuration. Then, during the implantation phase and in its service life, the device undergoes a characteristic loading sequence in which three different phases can be distinguished, and these are schematically depicted in Figure 1a. The stent is first subjected to a diameter reduction (i.e., crimping) to fit onto a catheter, it is maintained in such configuration through a sheath, and it is inserted into the human vascular system in a minimally invasive manner. Once it has reached the lesion site, the stent is released (i.e., deployment) and tries to recover its original dimensions thanks to PE; this guarantees the opening of the artery lumen and some structural support during the healing phase of the arterial wall. At this point, the stent is subjected to millions of working cycles due to blood pulsation and leg/head/heart movements [3], depending on its function, combining axial deformation, bending, and torsion [4] (Figure 1b).

The interest in investigating the response to cyclic loads of Ni-Ti stents has been motivated for many years [2], not only due to the intrinsic complexity of the topic but also especially due to the failures reported in clinics [5,6,7,8], which are connected with possible dramatic drawbacks, such as in-stent restenosis, pseudoaneurysms, and arterial perforation [9,10].

Fatigue is defined as the process of the progressive localized permanent structural change occurring in a material subjected to conditions that produce fluctuating stresses and strains at some locations; it may culminate in a fracture after a sufficient number of cycles at stress values that are far lower than the estimated ultimate strength [11]. Fatigue can be distinguished between low-cycle (LCF) and high-cycle fatigue (HCF) according to the deformation regime during loading: LCF is characterized by repeated plastic deformation, whereas HCF is characterized by elastic deformation. The number of cycles to failure is low for LCF and high for HCF, hence the nomenclature.

The mechanisms driving the fatigue of Ni-Ti alloys can be subdivided into functional and structural fatigue [12]. Functional fatigue refers to the progressive diminishing in functional properties during thermal or thermo-mechanical cycles (e.g., the dissipated energy from a pseudo-elastic damper or the stroke of a shape-memory actuator). Structural fatigue is given by microstructural damage accumulation: the defect, due to cyclic loads, propagates and reduces the cross-sectional area up to a limit value, after which the fracture abruptly happens.

During its working life, a stent cyclically deforms between two configurations, namely the fatigue peak and the fatigue valley (Figure 1a,b). The applied loads are perceived at the material level as a complex multiaxial state of cyclic stress and strain; it has been proven that even if a stent is loaded under a single cyclic axial load, the material’s nonlinearity together with the complex stent design is responsible for inducing a more complex response, where the local strain path might not be in phase with the applied remote load [13]. Indeed, even under uniaxial tension, a stent’s loading state is bending-dominated due to the configurations of the v-struts that result in it being subjected to transversal strain gradients in the cross-section (Figure 1c).

Before the commercialization of a new device, standard regulations prescribe experimental testing for evaluating the fatigue resistance of endovascular devices [15,16]. Tests-to-survive up to 10 million or 400 million cycles for stents and valve frames are required, respectively, to verify their durability in a scenario that is representative of 10 years of working life in vivo. This is an effective approach, but it is rather expensive both in terms of costs and time. Recently, computational evidence has been accepted by the regulatory authorities to support device marketing authorization, and this has led to the definition of ad hoc guidelines that are able to guide the user in the preparation of reliable virtual simulations [17,18]. From this, a different approach for fatigue analysis of Ni-Ti stents has been recently introduced involving the use of numerical simulations. After an experimental characterization of the device’s constituent material aimed at assessing its fatigue limit, computational simulations, first validated with a few fatigue-to-failure tests performed on the device, are then used to predict the outcomes of several loading conditions resembling in vivo cases, allowing for the verification and in-case optimization of the design [19], thus saving time and costs.

In this context, this review aims to enlighten the relevant choices that can affect the outcome of Ni-Ti stents fatigue analysis based on the coupling of experimental and numerical tests. In the selection of an in vitro setup, it is fundamental to consider the aspects impacting the device’s fatigue performance related to the Ni-Ti dependencies on the temperature and strain rate, as well as with the sample shape, dimensions, and thermo-mechanical treatments. On the other hand, from a numerical perspective, it is crucial to count on reliable models and adequate post-processing strategies based on ad hoc fatigue criteria that can synthesize the complex device’s internal state of stress and strain with a proper damage parameter.

The review is organized into two sections: the first analyses how the choice of different specimens, the testing environment, and frequency impact experimental results; then, the second section collects and discusses different choices in the preparation of simulations that can strongly affect the outcome of computational predictions.

## 2. Experimental Aspects Affecting Fatigue Analysis

**Figure 2:**
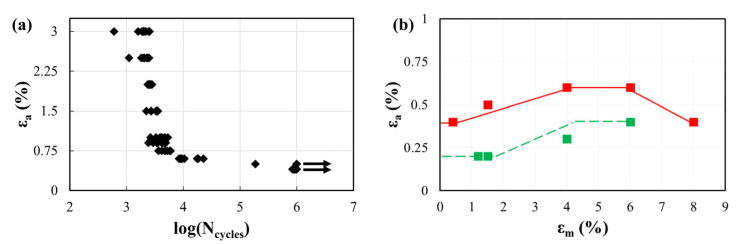
Results of a uniaxial tensile fatigue characterization performed on tensile wire specimens. (**a**) A strain–life diagram, performed at a fixed mean strain of 4%, where each point represents a failed sample; few samples reached the run-out of 1 million cycles and are represented by the arrow; (**b**) a constant-life diagram, considering the fixed threshold of 1 million cycles, where the solid line is an interpolation of the loading conditions leading to the failure of all samples (failure limit curve); the dashed line is an interpolation of the loading conditions leading to no failure (safe limit curve). Adapted with permission from [14] (Elsevier, 2023).

When dealing with the characterization of Ni-Ti material fatigue behavior, a strain-based approach is preferred to a stress-based one for performing in vitro tests due to the material’s non-linearities [20]. Even if the loading scenario of a stent is complex and mainly bending-dominated, it is quite common to perform uniaxial tensile tests or rotary bending tests on ad-hoc specimens, investigating different combinations of mean and amplitude strain values. In a standard fatigue analysis, these results are commonly reported in a strain–life diagram, where the strain amplitude is plotted against the number of cycles to failure (under a fixed mean strain value) (Figure 2a). However, a constant–life diagram is a more efficient way for Ni-Ti to highlight the role of mean strains on the fatigue behavior; by fixing the number of cycles to failure, the strain amplitude is plotted versus the mean strain (Figure 2b). In this way, by interpolating all the conditions leading to the sample’s failure, it is possible to obtain the material fatigue limit curve that shows a characteristic behavior with a maximum corresponding to about 5–6% of the mean strain.

In collecting such information, some aspects should be taken into account to obtain results representative of the material’s performance in vivo, as discussed below.

### 2.1. Type of Specimens

**Figure 3:**
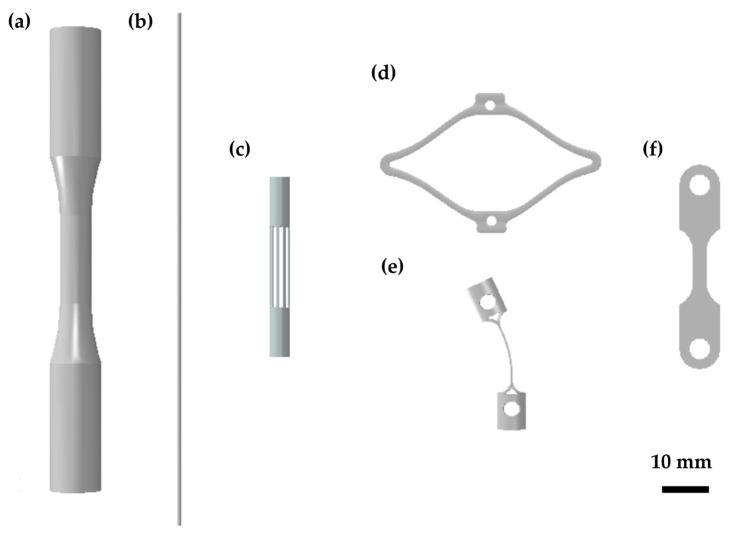
Graphical representation of the most common kinds of samples adopted for characterizing Ni-Ti fatigue behavior: (**a**) cylindrical bulk sample [21,22]; (**b**) single-wire specimen [24]; (**c**) laser-cut multi-wires sample [27]; (**d**) surrogate diamond-shaped sample [28]; (**e**) surrogate C-specimen [29]; (**f**) laser-cut macroscopic thin sample [31].

In the literature, the following different types of specimens (Figure 3) have been adopted for testing Ni-Ti fatigue performance, each with pros and cons:

- Bulk samples [21,22];
- Single wires [23,24,25];
- Laser-cut wires or multi-wire samples [26,27];
- Surrogate specimens [25,28,29];
- Macroscopic thin samples [30,31].

#### 2.1.1. Bulk Samples

Bulk samples are typically obtained from straight bars through a sequence of machining processes resulting in a cylindrical geometry with a uniform gage section, as required for low-cycle fatigue tests on common structural alloys. The specimens should be designed according to the ASTM requirements [32], with a gage section diameter lower than the grip section to ensure a predictable failure location (Figure 3a). Moreover, the overall dimensions must avoid buckling under fully reversed tests at a high strain amplitude. These samples allow for performing uniaxial fatigue tests (uniform strains in the cross-section) at different load ratios, including tension–tension, pulsating, and tension–compression conditions, by using a standard set-up including an extensometer for strain measurement. Additionally, the large size of the specimens permits the machining of flattened surfaces, which can be spray-painted to obtain a typical speckle appearance suitable for the digital image correlation (DIC) technique for measuring the strain field [33]. Despite the great advantages offered by bulk samples, the production process is significantly different from that adopted in the manufacturing of tubular forms for stents, resulting in a different microstructure [34], which can ultimately affect the crack formation process. Moreover, when considering large-sized bulk samples, the crack growth phase plays a fundamental role in the total fatigue life, with the macro-crack length exceeding the typical thickness of stent struts. Thus, the process leading to the final rupture in small and large Ni-Ti samples is different, and this discrepancy might lead to non-conservative fatigue life predictions [35].

#### 2.1.2. Single Wires

Single-wire samples are obtained by a proper manufacturing sequence including drawing as the last step to obtain a final diameter comparable to or slightly larger than the size of stent struts (Figure 3b). In this way, the macro-crack propagation effect on the fatigue life is removed. The fatigue behavior is commonly characterized by rotary bending fatigue tests under strain-controlled conditions. By using a proper set-up, the wire is rotated around a series of mandrels to induce a zero-mean strain and a strain amplitude on the surface inversely proportional to the radius of the curvature of the wire, allowing it to achieve a wide range of conditions by simply adjusting the bending curvature, with a strain gradient in the cross-section. By rotating the mandrels, the wire is subjected to tension–compression loading during each revolution. Given the simplicity of the experimental set-up and the high availability of wire samples, this technique has been widely used as a common method to characterize Ni-Ti fatigue [23,24,25]. However, the manufacturing process is not accurately representative of laser-cut devices, hence leading to an expected different microstructure that can affect the fatigue performance. Moreover, fully reversed loading at a load ratio of R = −1 (with R defined as the ratio between the minimum and the maximum load during a fatigue cycle) is not representative of the in vivo loading acting on the v-struts, with the load ratios ranging approximately between 0.2 and 0.7 considering the representative conditions of the study by Berti et al. [27]. Lastly, since in fully reverse loading the whole outer surface of the sample is repeatedly subjected to tension [25], this loading mode represents a worse condition with respect to a stent v-strut case, where the location of the tensile strains usually does not change dramatically during the cycle.

#### 2.1.3. Laser-Cut Wires

Another alternative is the use of laser-cut specimens obtained from the same tubes adopted in stent production and undergoing the same manufacturing process. This provides the great advantage of having samples with the same microstructure of stent struts characterized by a representative fatigue performance. Parallel wires can be machined along the tube circumference with a section comparable to that of stent struts (Figure 3c). A dog-bone geometry is typically adopted for tensile specimens with the radius in the fillet zone properly dimensioned to ensure the whole deformation is transferred to the wires. Since, in this case, and in the whole gage length, the cross-sections undergo uniform strains, a more conservative estimate of fatigue life is obtained. The main disadvantage of this geometry is the only possibility of performing tension–tension fatigue tests, paying attention to avoid those low-mean and high-amplitude conditions that can lead to the specimens buckling. This is in contrast to the complex multiaxial load applied in vivo, which is also perceived as a complex load path at the local scale of the stent strut [13].

#### 2.1.4. Surrogate Specimens

Alternatively, some authors have proposed the use of surrogate samples specifically designed to assess the Ni-Ti fatigue properties under strain-controlled conditions resembling the ones that cardiovascular devices experience in vivo. A well-known example is the diamond-shaped specimen introduced by Pelton and coworkers [28], resembling a single unit of a commercial stent geometry (Figure 3d). These sub-components are laser-machined from Ni-Ti tubes similar to those adopted in stent manufacturing to be more representative of the processing conditions, geometry, and material properties. A sequence of opening–closing cycles resembling the crimping, deployment, and fatigue loads of the device can be applied to evaluate the bending strain distribution over both the internal and external surface of the diamond arms, with the possibility of assessing the role of pre-straining on material’s fatigue performance. However, in most cases, these samples have scaled dimensions in terms of their strut length, width, and thickness; given the larger section, fatigue cracks can propagate with an increased path before reaching the critical length for fracture. For this reason, non-conservative fatigue life predictions might be obtained, as discussed. Moreover, finite element analysis (FEA) is required for the accurate computation of the strain levels from displacements, introducing errors associated with the numerical model itself and the adopted constitutive relations, aspects that are discussed in the following section. Within the category of surrogate samples, it is also worth mentioning the geometry developed by Cao et al. [29], who proposed a so-called C-specimen, a laser-cut wire from a thin-walled Ni-Ti tube with a shape set into a curved specimen (Figure 3e). In this case, pure bending is induced by displacing two pins mounted at the extremities toward each other. Even if FEA is still needed for evaluating the bending strains from the applied displacements, the simpler shape of the sample eases the simulations and also allows more accurate computations given the reduced strain gradients in the cross-section. However, the device is obtained from a straight laser-cut wire undergoing a shape-setting procedure that can introduce variability both in the real samples and, especially, when mimicked in a numerical model.

#### 2.1.5. Macroscopic Thin Samples

Other kinds of samples can be additionally found in the literature that are specifically designed to provide advantages according to the target application. These can be generally categorized as macroscopic thin samples, as only one of the characteristic dimensions is comparable to the size of the device strut. Moreover, a proper manufacturing sequence sharing some similarities with stent production is adopted to obtain representative microstructural features. To provide some examples, Catoor et al. [31] performed tension–tension fatigue tests on dog-bone samples that were laser cut from a thin Ni-Ti sheet (250 μm) with a macroscopic width of 2.6 mm (Figure 3f). Differently, Runciman et al. [30] adopted thin-walled tubes in manufacturing their stent (about a 3 mm outer diameter and a 250 μm thickness) to perform torsional fatigue tests at different strain ratios.

### 2.2. Testing Frequency and Environment

Regulatory bodies prescribe an experimental verification of cardiovascular devices for a number of loading cycles equivalent to 10 years of in vivo implantation by adopting a test-to-survive approach. Accounting for heart beating and typical gait cycle frequencies, fatigue cycles at a loading rate of roughly 1 Hz can be estimated on these devices, which would require about four months and a decade of testing for peripheral stents and valve frames, respectively. As such tests would be extremely time-consuming and not affordable in the verification and validation phase, accelerated durability tests are typically performed in the design phase by applying a loading frequency of up to 50 Hz [26,28,36]. However, besides the fact that it may be desirable to perform the tests as quickly as possible to obtain the results in a reasonable time, the influence of the loading frequency in the context of Ni-Ti testing represents a complex theme that has to be carefully addressed.

**Figure 4:**
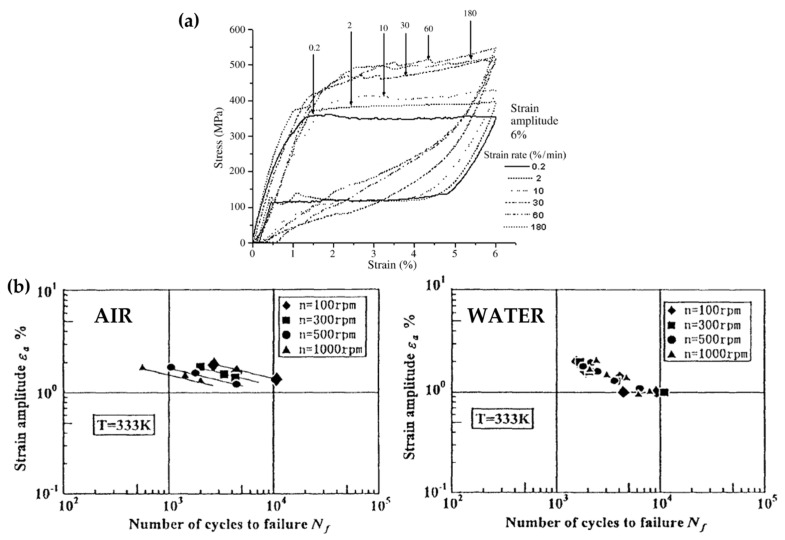
(**a**) Strain rate sensitivity of the stress–strain response of Ni-Ti, reprinted with permission from [43] (Elsevier, 2023). (**b**) Comparison between the strain–life plots at different rotational speeds in air and water, reprinted with permission from [46] (Elsevier, 2023).

Fatigue testing in an air environment represents an advantageous choice that has adopted by many research groups both for device testing [14,37] and material fatigue characterization [28,29], as it allows for simplifying the experimental setup and adopting both optical techniques for strain measurement [38] and infrared thermography for temperature monitoring [39]. However, a heating effect associated with the latent heat generated during phase transformation is well-recognized in air that can ultimately affect the characteristic stress–strain response of the material with a strong influence on the testing frequency [40,41,42]. It is well-established that at quasi-static strain rates, comparable to those at which static material characterization is performed (below 10^−4^ s^−1^), the latent heat generated during stress-induced martensitic transformation can be timely exchanged with the surrounding environment without any increase in the temperature of the specimen, leading to a characteristic flag-shaped stress–strain curve [43,44] (Figure 4a). As the loading rate increases from 10^−4^ s^−1^ to 10^−3^ s^−1^, the available time for latent heat convection decreases, causing a slight increase in the temperature of the specimen during loading and a decrease during unloading [39,40,42,44]. Given the well-known Clausius–Clapeyron relation, establishing a linear relationship between the temperature and the transformation stresses [45], this heating effect determines a slightly higher forward transformation stress and a reduced reverse transformation stress, as is visible in Figure 4a. By further increasing the loading frequency up to high strain rates (above 10^−3^ s^−1^), the temperature of the sample drastically changes during the phase transformation as the latent heat cannot be convected timely. Heat accumulation inside the material causes a hardening effect with a progressive increase in the slope of the stress plateaus [40,41,42]; moreover, as heating provides a driving force for transformation during unloading, reverse transformation from martensite to austenite occurs at higher stresses, and the hysteresis loop narrows [44]. According to a recent work by Sidharth et al. [44] investigating the damping capacity of differently oriented Ni-Ti single crystals in a wide frequency range between 0.01 Hz and 10 Hz, a stabilization of these effects seems expected, even if data regarding common testing frequencies for stent durability have not been provided. Specifically, they attributed the damping capacity associated with Ni-Ti stress hysteresis to the complementary action of three mechanisms, among which the friction at the austenite/martensite interface was recognized as providing the major contribution.

The heating effect in the air was additionally found to influence the fatigue life, with a reduction in the number of cycles to failure being associated with an increased loading rate [46]. A possible solution to the discussed issues is performing fatigue testing in a liquid environment following the suggestions of the Food and Drug Administration (FDA) [16]. In support of this choice, many authors refer to the work of Tobushi et al. [46] who, when performing rotary bending fatigue testing on wire samples in water, observed an insensitivity of the fatigue life to the testing frequency associated with the high heat transfer coefficient of the adopted liquid mean. However, the translation of such evidence to device testing is not straightforward since rotary bending is related to a different solicitation of the struts in comparison to the in vivo loading scenario, and only the fatigue cycles at the beginning of the hysteresis cycle (i.e., those associated with limited phase transformation) were investigated by the authors (Figure 4b). Other research groups investigating the Ni-Ti behavior under accelerated loading confirmed the improved heat transfer phenomenon in a liquid environment, but they still observed an effect of the loading rate on the characteristic response of the material [31,47]. Specifically, Catoor et al. [31], when applying uniaxial cyclic loads on dog-bone samples, reported similar effects of the testing frequency on the Ni-Ti stress–strain curve to those discussed for the air environment. Shaw et al. [47], when performing a single loading–unloading cycle on Ni-Ti polycrystalline wires in both water and air, reported a non-negligible heating of the sample even in water (to a lower extent with respect to air), additionally assessing an enlargement of the hysteresis loop with the frequency in contrast with the observations of Sidharth et al. [44] on single crystals. The absence of consistency among the various literature data suggests that the influence of the environment and the loading frequency on Ni-Ti behavior is actually associated with a multiplicity of factors including the microstructure and loading conditions.

From this discussion, it is possible to state that the influence of the testing frequency on Ni-Ti fatigue behavior is mitigated in water with respect to air, even if it cannot be completely neglected. It is reasonable that the impact of the testing frequency on fatigue life in water should be lowered too, but the intrinsic variability of the fatigue testing has to be taken into account in such considerations. The optimal approach for avoiding any uncertainty would be performing fatigue testing of Ni-Ti devices and components at low frequencies in a liquid environment, which would drastically increase the cost of the experimental phase. Moreover, when adopting in silico models, the material’s rate-dependency should be taken into account to obtain an accurate computation of the stresses and strains in the various locations of the strut, thus extremely complicating the constitutive formulation [48].

## 3. Numerical Aspects Affecting Fatigue Analysis

The great advantage of using in silico tools for fatigue analysis is the possibility to know the stress and strain tensors at each location of the device. The model must be built to reflect the real device, both in terms of its design and mechanical behavior; this guarantees the credibility of the results, and therefore it is important to recognize the relevant aspects affecting the global and local mechanical properties. Indeed, the geometric complexity of the device’s structural units, together with the material’s nonlinearity, is responsible for a three-dimensional stress/strain that is also characterized by gradients, making the prediction of the fatigue behavior extremely challenging.

### 3.1. Model Reliability

In the last years, there has been increasing scientific interest in the definition of good practices for the preparation of credible models [18,49,50], and there are many examples of Ni-Ti stents with in silico models used for investigating their static and fatigue performances [51,52,53,54]. Among others, two key aspects have to be carefully considered, namely the device’s geometrical features and the material’s properties. If the activity is conducted in collaboration with the manufacturer, it is possible to have easier access to an accurate device CAD, accounting for laser cutting and finishing treatments that might affect the dimensions of the struts, and the exact material behavior, which otherwise is not available to those researchers willing to exploit numerical tools for analyses. Indeed, in most cases, the geometry is reconstructed through optical observation [7,55,56]; this is quite an established and reliable method, which is also simplified by the presence of repetitive units in the stent cells. However, this can introduce errors depending on the quality of the acquired measurements; even a discrepancy of a few microns can alter the simulation results.

On the other hand, the knowledge of the mechanical properties of the constituent material remains the most challenging task, especially in the case of Ni-Ti alloys that exhibit complex non-linear and temperature-dependent behavior, requiring many parameters to be known. The most common approach for preparing such simulations is to take the Ni-Ti material parameters from the literature [24,52,54,57,58] without having the possibility of testing the material. When referring to medical-grade Ni-Ti alloys, the percentage Ni content is quite stable and might slightly vary (typically between Ni_50.6_Ti_49.4_ and Ni_51_Ti_49_), resulting in minor variations in terms of mechanical properties. However, the major impact comes from the proprietary heat treatment procedures that each manufacturer performs on the source tubes for guaranteeing proper transformation temperatures and adjusting the mechanical behavior [59].

**Figure 5:**
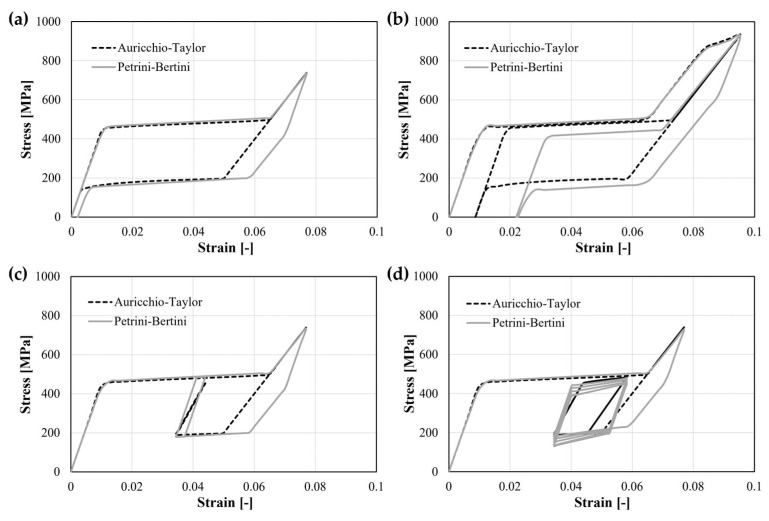
Comparison between the results of two different constitutive models formulated for describing Ni-Ti alloy behavior. (**a**) Superelastic loading and unloading; (**b**) superelastic–plastic loading, followed by total unloading, showing residual strain, and a second loading phase; (**c**) superelastic loading and partial unloading, followed by fatigue cycles with low amplitude strains; (**d**) superelastic loading and partial unloading, followed by fatigue cycles with higher amplitude strains, where it is possible to appreciate the different behavior related to the inelastic strain accumulation.

Even assuming that the exact material properties are perfectly known from experiments, the choice of the constitutive model used for simulating such behavior introduces the need of accepting some approximations. Indeed, there is a trade-off between the degree of realism that the material model can reach for describing real phenomenology and the usability of such a model. There are many examples in the literature of user subroutines that have been developed ad hoc to describe thermo-mechanical Ni-Ti behavior in a very realistic way (e.g., including superelastic–plastic effects, inelastic strain accumulations due to fatigue, shape memory), but these require a considerable number of input parameters, and, consequently, many experiments for their assessment [60,61,62]. Commercially available software for structural simulations, such as Abaqus (Dassault Systèmes) and Ansys Mechanical (ANSYS), exploit the Auricchio–Taylor constitutive model [63] for superelastic materials; it is able to catch the most important aspects of PE, requiring the knowledge of only seven parameters that are easily detectable on the material’s tensile curve. However, consequently, it approximately describes some behaviors, such as the effect of plastic strain accumulation during loading, as shown in Figure 5, where the Auricchio–Taylor [63] and the Petrini–Bertini [61] models, calibrated with a comparable set of parameters and tested under the same boundary conditions, are compared; even under superelastic loading and unloading there are differences in the description of the curve (Figure 5a), which become notable when plastic phenomena are included (Figure 5b) and also inelastic strain accumulation due to fatigue (Figure 5c,d). Moreover, the Auricchio–Taylor model takes into account the tension/compression asymmetry that characterizes Ni-Ti stress–strain curve in a very simplified manner, and this strongly affects the strain gradient in bending, scaling the tensile response just through one parameter (that is related to the ratio between the stress at which the transformation from austenite to martensite starts in compression and tension).

### 3.2. Simulation of Fatigue Tests

**Figure 6:**
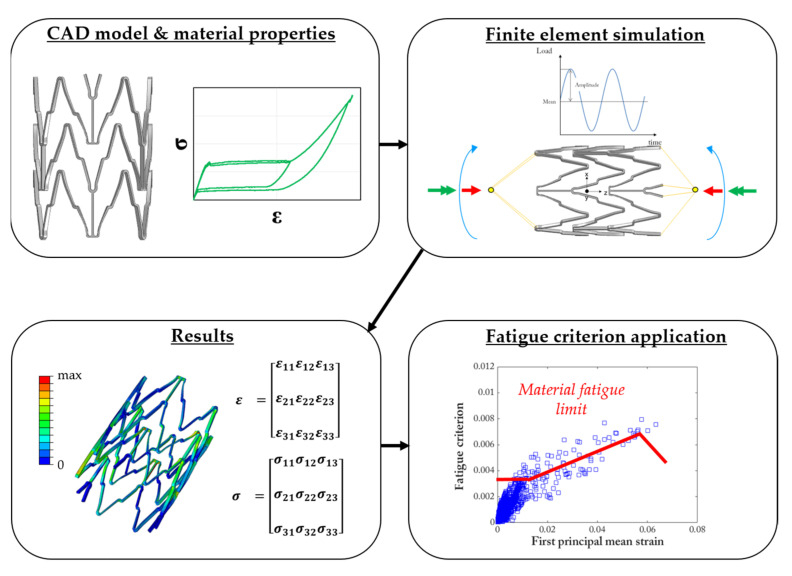
In silico workflow for the fatigue analysis of a Ni-Ti device. Starting from the knowledge of the design and material properties, proper cyclic boundary conditions are applied to the device to assess the local stress and strain quantities at each location of the model. Then, each element is evaluated through a proper fatigue index (represented through a blue box in the plot) to discriminate between safe and critical conditions with respect to the material’s fatigue limit (red line in the plot).

Figure 6 summarizes a typical workflow for the realization of a computational fatigue analysis of Ni-Ti stents, where the numerical model of the device, which at this stage has proven reliable, is subjected to a representative set of loads in the form of a few cycles to allow numerical stabilization. When in silico tools are used in support of experiments, it is crucial to be consistent in the application of the boundary conditions: for this reason, the aim of those interested in performing a coupled numerical–experimental analysis is to deal with controllable and easily reproducible experiments [18]. At this stage, numerical outputs such as local stress and strain fields are extracted and properly managed to interpret fatigue failures.

Taking into account the latest European technical documentation on the matter of endovascular devices [64], the in vitro fatigue verification of a newly designed stent should involve a replica of the cyclic loading conditions that represent the in vivo environment. This requires starting from the device deployment into mock tubes to mimic the device’s radial constraint due to the vessel. For the previously discussed Ni-Ti-related issues, a liquid should be circulating in the system for promoting heat exchange and maintaining a constant temperature. Then, for example, dealing with peripheral stents, it is recommended to perform fatigue campaigns by applying axial loads, bending, torsion, and lateral compression to the tubes, which, in turn, transfer to the stent [36,65]. In the standards, there is no explicit reference to the simultaneous application of such loads, which would require more sophisticated testing machines.

While these tests are quite an established procedure for those willing to follow the test-to-survive approach, such experiments are characterized by a high degree of uncertainty that hinders their direct use as comparators of numerical studies. Indeed, the presence of the tube plays a crucial role in affecting the initial state of the stent, albeit in a difficultly predictable way. First, it would be necessary to conduct a thorough characterization of the tube’s constituent material (usually silicone or latex) for assessing its radial compliance, which is the key element in defining the equilibrium configuration at the end of the deployment [66], thus, the initial stress and strain state in the stent before cycling can be obtained [67]. Then, since remote loads are applied to the tubes, a fine calibration of the stent–tube friction properties should be addressed for a reliable description of the load quota transferred to the stent during the test.

For these reasons, in the literature, most of the in vitro studies that are replicated in silico involve fully-expanded Ni-Ti stents subjected to single or multiple loads directly applied at the device’s extremities [14,26,68]. These conditions are not strictly representative of the in vivo scenario, but the use of simulations can guide the selection of the worst cases usable for testing such devices to failure.

### 3.3. Fatigue Criteria

To produce in silico predictions, standard regulations [64] prescribe the use of simulations for the evaluation of stresses and strains in the device that must be compared to the material fatigue limit to calculate a fatigue safety factor. A recurrent choice in the fatigue analysis of Ni-Ti stents is based on the assumption that the strains in the device do not change orientation during the cycle, as in uniaxial tensile or pure bending tests. In this way, the strain tensors are evaluated at the maximum and minimum values of the cycle, namely the fatigue peak and valley. Then, by performing an algebraic sum/difference, it is possible to calculate the mean and amplitude strain tensors at each location of the model, from which the principal values are deducted; these can be directly evaluated in a constant–life diagram with respect to the material fatigue limit [53,69]. Another possibility is to re-interpret the amplitude strain tensor according to the definition of the von Mises equivalent strain, which suggests improperly defining this approach as the “von Mises fatigue criterion” [30,70]. The von Mises (*VM*) index is calculated by using strain tensors extracted from the two time increments corresponding to the peak and valley of the fatigue cycle. The difference between the two tensors is calculated for obtaining the alternate strain tensor, from which the alternate values of the principal strains are derived, namely Δ*ε*_1_, Δ*ε*_2_, an Δ*ε*_3_ (Equation (1)), and these are combined using a scaling factor including the Poisson’s ratio value, *ν*, of the material:

(1) $$VM=\frac{1}{2\sqrt{2}(1+\nu )}\sqrt{{\left(∆\varepsilon_{1}-∆\varepsilon_{2}\right)}^{2}+{\left(∆\varepsilon_{2}-∆\varepsilon_{3}\right)}^{2}+{\left(∆\varepsilon_{3}-∆\varepsilon_{1}\right)}^{2}}$$

This is quite an effective but simplified approach that neglects the presence of a three-dimensional stress/strain state in the stent, as per in vivo conditions. Indeed, even under a single uniaxial remote load, the strains experienced by each element of the stent model change orientation, exhibiting both normal and shear components [13]. In this case, the local strain path defined during the cycle is defined as non-proportional, and the information at the fatigue peak and valley is not sufficient for the accurate description of the local quantities acting in the device. More sophisticated methods may be required for the synthesis of the local quantities to be associated with fatigue failure in such conditions [71].

In the literature, many criteria have been formulated for interpreting fatigue data in cases of standard metals loaded non-proportionally: these are based on the concept of the critical plane, that is, the specific plane at the material point level on which a local quantity is maximized during the whole cycle (and not only considering the peak and valley instants, as in the uniaxial or proportional cases) [72]. This local quantity may change according to each criterion (e.g., the shear amplitude strain or the normal strain), and it is addressed as the primary cause for fatigue crack nucleation and propagation.

The Fatemi–Socie (*FS*) parameter [73] is a strain-based critical plane criterion for shear-failure-mode materials, which is a function of both the maximum shear strain amplitude (Δ*γ_max_*/2), which is assumed to cause the crack formation, and the maximum value of normal stress (*σ_n,max_*), which is responsible for the crack propagation in tension, both acting on the plane of the maximum shear strain amplitude (Equation (2)):

(2) $$FS=\frac{∆\gamma_{max}}{2}\left(1+k\frac{\sigma_{n,max}}{G∆\gamma }\right)$$

The herein-reported fatigue parameter *G* Δ*γ* (Δ*γ* is the shear strain range on the maximum shear strain plane) has been recently proposed in the work of Gates and Fatemi [74] as an update to the original fatigue parameter that included the material yield strength. The material parameter *k* acts as a scaling factor that can be determined by correlating axial and torsion data. This new version of the *FS* criterion has been proven to better correlate fatigue data in the presence of mean stress, even though it maintains all the advantages and physical interpretations of the original criterion [75].

The Brown–Miller (*BM*) parameter [76] is also a strain-based criterion that combines the maximum shear strain amplitude, which is assumed to cause the crack initiation, and the maximum normal strain amplitude (Δ*ε_n_*/2), associated with crack propagation, which occurs in a cycle on the plane experiencing the maximum shear strain amplitude (Equation (3)):

(3) $$BM=\frac{∆\gamma_{max}}{2}+S\frac{∆\varepsilon_{n}}{2}$$

*S* is a material-dependent parameter that represents the influence of the normal strain on material crack growth, and it is determined by correlating axial and torsion data. The definition of the shear strain amplitude is not straightforward when dealing with multiaxial loads, and different methods have been proposed in the literature [71,77,78].

The Smith–Watson–Topper (*SWT*) parameter [79] is an energy-based approach where the fatigue life is assumed to be dominated by crack initiation and growth along the plane of maximal principal strain. The fatigue index is given by the product of the maximum normal stress and the maximum normal strain amplitude (Equation (4)), as in the form reported by Socie [80]:

(4) $$SWT=\sigma_{n,max}\frac{∆\varepsilon_{n}}{2}$$

Calhoun et al. [81] used the SWT for the lifetime prediction of shape-memory alloys undergoing thermal cycling and obtained good results in the interpretation of experimental data.

**Figure 7:**
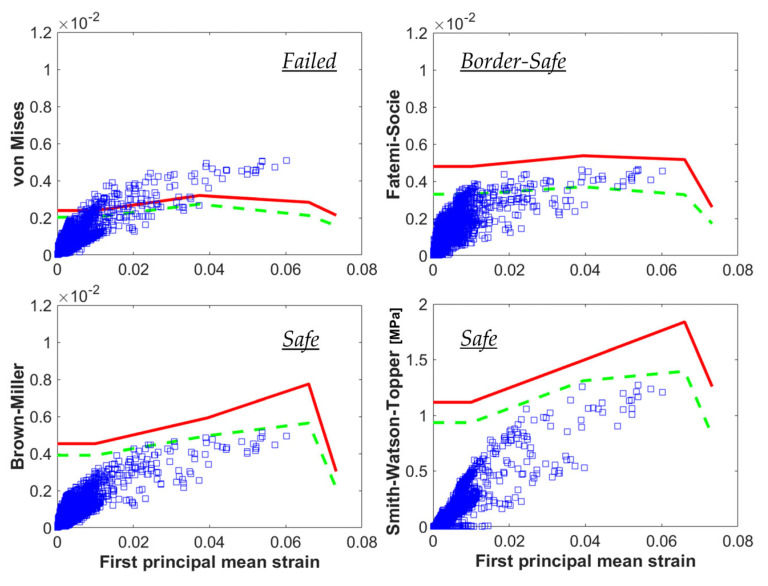
Results of a coupled in vitro and in silico study on the fatigue behavior of peripheral stents, adapted with permission from [14] (Elsevier, 2023); considering the same experimental outcome (safe), the use of different fatigue criteria for calculating the parameters for each element of the numerical model (blue boxes) led to different predictions (i.e., different distances from the material limit curves, in green and red), proving a better accuracy of the critical plane-based approaches compared to the von Mises.

In the work of Berti et al. [14], these approaches have been compared for interpreting experimental failures in multiaxial campaigns on peripheral stents. For each criterion, a constant–life chart was built with the specific fatigue index plotted against the first mean principal strain. Then, the experimental information regarding the material fatigue strength was translated into a uniaxial limit curve, separating the plots into a safe area and a critical area for failure. As expected, the critical plane-based indices correlated the fatigue data better than the von Mises (Figure 7a).

A different approach is the one based on the Dang Van-type criterion for shape-memory alloys proposed in the work of Auricchio et al. [82]. This criterion assumes that fatigue damage is controlled by mechanisms at the grain scale in the polycrystalline material. In particular, it is based on the concept of shakedown, which in shape-memory alloys depends on the formation of the stress-induced martensite volume fraction, the transformation and reorientation strain values, and/or the occurrence of transformation-induced plasticity in the fully martensitic state. Accordingly, several material responses can be identified, for example, elastic shakedown (this can only be associated with a high-cycle fatigue life), alternating phase transformations, alternating plasticity, or ratcheting. The Dang Van (*DV*) fatigue damage parameter can be expressed as (Equation (5)):

(5) $$DV=\underset{t>t_{0}}{\max}\frac{\hat{\tau }\left(x,t\right)+a\left(\alpha \left(x\right)\right)\hat{\sigma_{h}}\left(x,t\right)-b(\alpha \left(x\right))}{b(\alpha \left(x\right))}$$

where $\hat{\tau }$ and $\hat{\sigma_{h}}$ are, respectively, the shear and hydrostatic stress at the mesoscopic scale, a and b are parameters that depend on the transformation-induced variable *α*, which, in the absence of experimental data, can be assumed to be the martensite volume fraction. Positive values of the parameter indicate failure, while negative values indicate an infinite lifetime. However, the only literature study using this approach for the computational fatigue analysis of Ni-Ti stents [83] found that the predictions were consistent with the von Mises approach and the one based on the construction of a principal strains constant–life diagram. This might be due to some approximations on the use of the literature data for the material parameters a and b.

The choice of one of the aforementioned multiaxial criteria should assume the knowledge of the specific fatigue failure mode of the material. However, at present, there is no consensus on the Ni-Ti damage mechanism during cyclic loading in different conditions (fully austenite, fully martensite, mix of phases). One reason for this lack in the literature is represented by the experimental difficulty in accurately tracking crack growth in such small components. Moreover, once a macroscopic crack enucleates, there is a short residual time before the structural failure of the device. In these cases, a total life approach is deemed to be appropriate. A damage tolerance analysis can still be used as a life prediction approach to quantify the reduction in performance due to defects [27], but in general, it is easier to directly correlate the in-service performance of the device with a total fatigue life “benchtop” prediction [84].

## 4. Conclusions

The fatigue analysis of Ni-Ti endovascular devices is a multifactorial research topic that requires careful consideration for obtaining credible results. In particular, we summarize the main considerations discussed in this work as follows:

- Material fatigue characterization can be performed using different samples, whose design also inevitably affects the testing configuration (e.g., rotary bending, axial tension/compression, pure bending, etc.). This, in turn, leads to the generation of material fatigue limit data that should be discussed before use; for example, results from testing wire specimens overestimate the failure risk of a stent v-strut loaded under the same maximum strain. The samples that are closer to stents in terms of their geometry, strut dimension, manufacturing process, and loading conditions are the surrogate diamond-shaped samples, which, however, mandatorily require a finite element model to assess the local stress and strain;
- The need for accelerated durability tests opens questions regarding the material strain rate sensitivity, which alters the stress–strain behavior due to the intrinsic heat of the sample due to the fast and repeated lattice transformation. Standard regulators suggest performing tests in a liquid environment to allow a better heat exchange and to reduce such effects, which, however, cannot be discarded when working at values of tenths of a Hz;
- When preparing a device computational model, it is crucial to work with an accurate geometry associated with proper mechanical properties. However, due to the bending-dominated deformation of the v-struts, even minor discrepancies in the model might introduce sensible differences in the global behavior. Moreover, different constitutive formulations for shape-memory alloys are available in the literature that are able to differently account for the complex material phenomenology, thus, affecting the numerical outcomes;
- The choice of easily reproducible and controllable experiments is of paramount importance for comparing simulation results with the reality of interest;
- For conducting a computational fatigue analysis, a proper fatigue criterion should be selected for the interpretation of the local tensors of stress and strain. There are no available criteria specifically formulated for Ni-Ti and shape-memory alloys, and the choice of different approaches might lead to different outcomes. It is recommendable to prefer those criteria that are able to account for complex multiaxial loading conditions, which are known as critical plane-based criteria.

## Data Availability

Data sharing is not applicable to this article as no new data were created or analyzed in this study.
